# Human Papillomavirus (HPV) and HPV Vaccine Awareness Among U.S. Adults With Depression and Anxiety: A Nationally Representative Analysis Using Health Information National Trends Survey (HINTS) Data

**DOI:** 10.7759/cureus.95218

**Published:** 2025-10-23

**Authors:** Akinyemi Akinwumiju, Ifeoma Amadi, Fola Ishola, Benedette C Okonkwo, Chinelo Onochie, Bibobra Tagbatsemi, Azeez Oseni, Obianuju Nwauwa, Diana Mensah

**Affiliations:** 1 Psychiatry and Behavioral Sciences, Progressive Psychiatric Services, Las Vegas, USA; 2 Family Medicine, Valley Health System, Las Vegas, USA; 3 Healthcare, University of Southern Mississippi, Houston, USA; 4 Medicine, Northridge Hospital Medical Center-Optum Insight, Los Angeles, USA; 5 Family Medicine, Jefferson Einstein Philadelphia Hospital, Philadelphia, USA; 6 Clinical Research, University of California Davis Health System, Sacramento, USA; 7 Family Medicine, El-Roi Multiclinic and Reprocare, Abuja, NGA; 8 General Medicine, Windsor University School of Medicine, Cayon, KNA; 9 Family Medicine, Nsawam Government Hospital, Accra, GHA

**Keywords:** anxiety, depression, health disparities, health literacy, hints, hpv, hpv vaccine, public health

## Abstract

Background

Depression and anxiety are highly prevalent in the U.S. and can hinder engagement in preventive health services. Individuals affected by these conditions may have reduced motivation and lower health literacy, increasing their vulnerability to missed preventive-care opportunities such as vaccination. Awareness of human papillomavirus (HPV) and the HPV vaccine is crucial for cancer prevention but remains underexplored among adults with mental-health conditions.

Methods

We analyzed data from the Health Information National Trends Survey (HINTS) 5, Cycles 1-4 (2017-2020), to assess HPV and HPV-vaccine awareness among U.S. adults aged 18-44 with self-reported depression and/or anxiety. Weighted descriptive statistics, chi-square (χ²) tests, and multivariable logistic-regression models were used to assess awareness and associated sociodemographic and health-related factors. The primary aim was to estimate the prevalence of HPV and HPV-vaccine awareness, while the secondary aim was to identify associated sociodemographic and health-related predictors. Awareness was defined using binary (“yes”/“no”) responses to HINTS items, and mental-health exposure was defined as self-reported physician-diagnosed depression and/or anxiety.

Results

The analytic sample included 1,105 adults, most of whom were female (n = 709; 64.1%), aged 18-34 years (n = 623; 56.4%), White (n = 753; 68.2%), and urban residents (n = 956; 86.5%). Approximately one-third (n = 372; 33.5%) held a college degree, and n = 384 (34.8%) reported annual income ≥ $75,000. Overall, 944 (85.4%) were aware of HPV, and 839 (75.9%) were aware of the HPV vaccine. Higher educational attainment (χ² = 46.7, p < 0.001) and female sex (χ² = 6.88, p = 0.009) were associated with greater awareness, whereas Hispanic ethnicity (χ² = 9.4, p = 0.024) and male sex were linked to lower awareness. Participants with two or more comorbidities showed higher HPV awareness (χ² = 6.11, p = 0.047) but not vaccine awareness (χ² = 2.34, p = 0.311).

Conclusions

While overall awareness of HPV is high, disparities persist by sex, education, and ethnicity among adults with depression and/or anxiety. Integrating culturally tailored and literacy-appropriate HPV education into mental-health and primary-care settings may promote equitable vaccine awareness and uptake.

## Introduction

Mental-health disorders, particularly depression and anxiety, represent a significant public-health concern in the U.S. Recent estimates indicate that approximately 8.3% of U.S. adults experience at least one major depressive episode annually, with higher prevalence among women (10.3%) compared with men (6.2%) [1]. Anxiety disorders are even more common, affecting 19.1% of adults each year [2]. These conditions not only cause significant individual suffering but are also associated with diminished quality of life, impaired functioning, and increased risk of co-occurring physical health conditions [3]. Notably, they are especially prevalent among young adults aged 18-25 years, a demographic that also falls within the recommended age range for human papillomavirus (HPV) vaccination [1,2].

HPV infection and its associated cancers constitute another major public-health challenge. As the most common sexually transmitted infection in the U.S., HPV is a primary cause of cervical, anal, oropharyngeal, penile, vulvar, and vaginal cancers [3,4]. Globally, HPV accounts for a substantial proportion of infection-related cancers. The HPV vaccine provides highly effective primary prevention, capable of preventing up to 90% of HPV-related malignancies [4,5]. Broad vaccine uptake, especially during adolescence, is therefore central to reducing the HPV-associated cancer burden [4,5].

Despite its proven efficacy, disparities in HPV awareness and vaccination persist, particularly among underserved and marginalized populations. Lower socioeconomic status, minority racial or ethnic background, limited access to care, and reduced health literacy are well-documented barriers [6,7]. Individuals living with depression or anxiety may constitute another vulnerable subgroup. Psychiatric symptoms, such as low motivation, cognitive slowing, and social withdrawal, can hinder engagement with preventive health behaviors [7,8]. Depressive and anxious individuals are also less likely to maintain regular healthcare visits or seek preventive services, which may limit opportunities to learn about vaccines. Moreover, an HPV diagnosis itself can trigger psychological distress, further reinforcing the bidirectional link between mental health and HPV-related outcomes [9].

While HPV awareness has been widely studied in the general population, little is known about awareness among adults with mental-health conditions. Understanding this relationship is crucial, as impaired information processing, stigma, and healthcare disengagement may reduce exposure to preventive information. Prior research on other topics (e.g., clinical-trial participation) has shown that individuals with mental-health conditions, especially those with lower educational attainment or without regular providers, tend to exhibit lower awareness and knowledge levels [10]. These findings suggest that similar patterns may exist for HPV awareness and vaccination.

The primary aim of this study is to estimate the prevalence of awareness of HPV and the HPV vaccine among U.S. adults aged 18-44 years with self-reported depression and/or anxiety. The secondary aim of this study is to identify sociodemographic and health-related factors associated with HPV and HPV vaccine awareness in this population. By clarifying these aims and integrating a behavioral-health perspective, this study extends prior HPV awareness research to a high-risk subgroup often overlooked in preventive-care initiatives. These findings may inform the development of culturally and literacy-appropriate public-health strategies that integrate preventive education within mental-health and primary-care settings.

## Materials and methods

**Figure 1:**
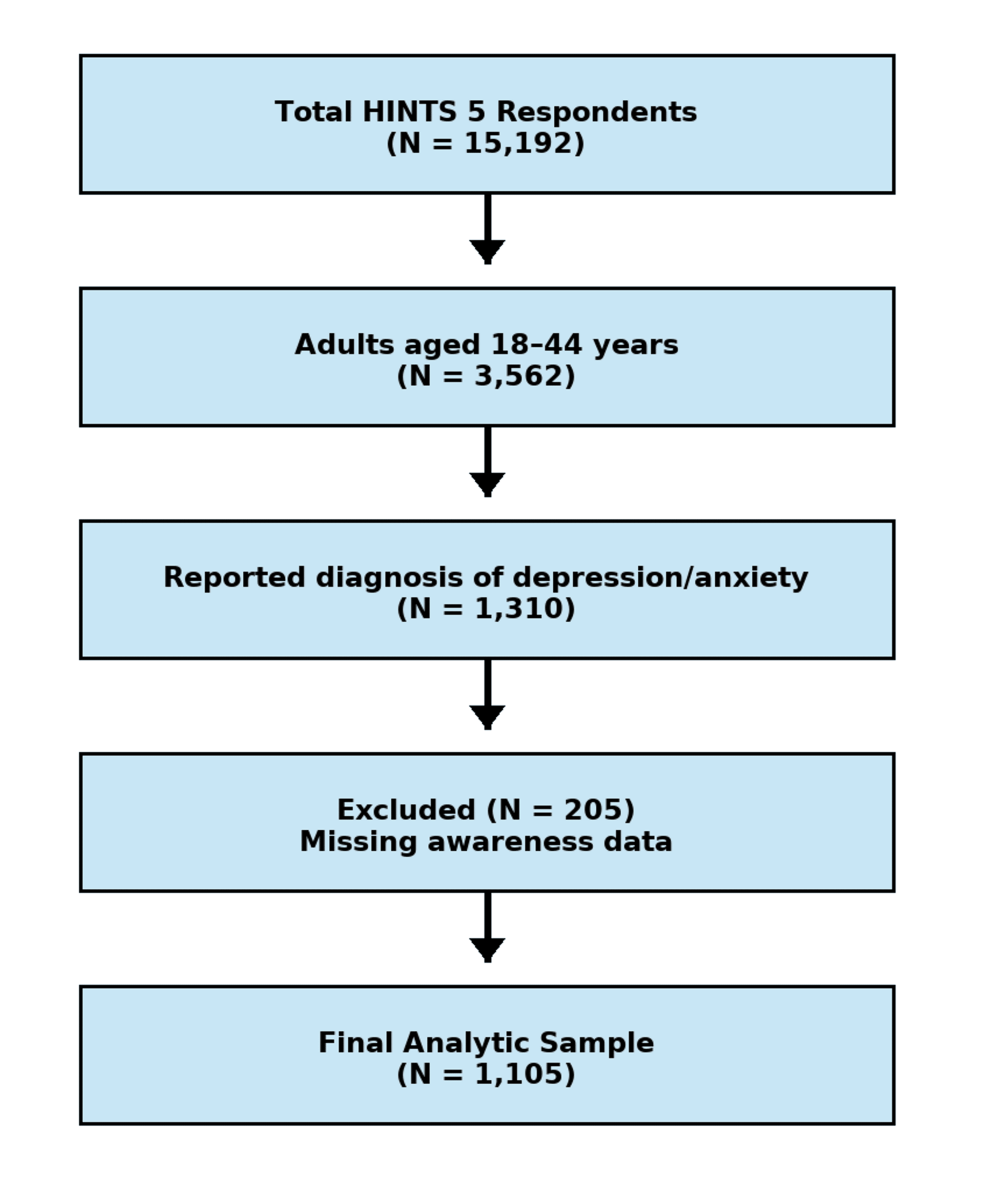
Study flow diagram illustrating participant selection from the Health Information National Trends Survey (HINTS) 5, Cycles 1-4 (2017-2020). Of 15,192 total respondents, 3,562 were aged 18-44 years, 1,310 reported depression and/or anxiety, and 1,105 were included in the final analytic sample after excluding 205 participants with missing responses for HPV or HPV vaccine awareness items. Data represented as N (%). Statistical significance is considered at p < 0.05. Chi-square (χ²) values are used for group comparisons.

This cross-sectional study followed the Strengthening the Reporting of Observational Studies in Epidemiology (STROBE) reporting guidelines and utilized publicly available data from the Health Information National Trends Survey (HINTS) 5, Cycles 1-4 (2017-2020). HINTS is a nationally representative mail-based survey administered by the National Cancer Institute to assess how U.S. adults access, understand, and use health information. It targets the civilian, non-institutionalized adult population through a two-stage stratified sampling design. Response rates across Cycles 1-4 ranged from 30.3% to 36.7%. Analyses were restricted to respondents aged 18-44 years who reported a diagnosis of depression and/or anxiety. Weighting variables provided by HINTS were applied to ensure population representativeness. A study flow diagram (Figure 1) illustrates the inclusion and exclusion process: of 15,192 total respondents, 3,562 were aged 18-44 years, 1,310 reported depression and/or anxiety, and 1,105 were included in the final analytic sample after excluding 205 participants with missing responses for HPV or HPV vaccine awareness items.

All participants provided written informed consent prior to participation. The HINTS protocol was approved by the Westat Institutional Review Board. Because the dataset is deidentified and publicly available, this secondary analysis was considered exempt from additional review by the NIH Office of Human Subjects Research Protections. The primary outcomes were awareness of HPV and awareness of the HPV vaccine. HPV awareness was determined by responses to the question, “Have you ever heard of HPV?” and HPV vaccine awareness was assessed by, “Have you ever heard of the cervical cancer vaccine or HPV shot?” Responses were binary (“yes” = 1, “no” = 0). The key independent variable, mental health exposure, was defined as self-reported physician-diagnosed depression and/or anxiety based on the question, “Has a doctor or other health professional ever told you that you had depression or anxiety disorder?”

Covariates included demographic and health-related variables such as age (18-34, 35-44), sex (male, female), race/ethnicity (White, Black, Hispanic, Other), education (high school or less, some college, college graduate or higher), household income (<$50,000, $50,000-$74,999, ≥$75,000), marital status (married/partnered vs. single/divorced/widowed), health insurance (insured vs. uninsured), regular healthcare provider (yes/no), smoking status (current, former/never), medical comorbidities (none, one, ≥2), and residence (urban vs. rural). Comorbidities were based on self-reported diagnoses of diabetes, hypertension, heart disease, cancer, or lung disease. Participants with missing data on HPV or HPV vaccine awareness (n = 205) were excluded. For covariates, missing responses constituted less than 5% of the sample and were handled through listwise deletion to maintain internal consistency across analyses.

All analyses were conducted using Stata version 17.0 (StataCorp LLC, College Station, TX). The svy suite was used to account for the complex survey design and sampling weights. Weighted descriptive statistics summarized sample characteristics as means ± standard deviations (SD) for continuous variables and weighted frequencies and percentages (N, %) for categorical variables. Chi-square (χ²) tests assessed bivariate associations between categorical predictors and awareness outcomes, with test statistic values and p-values reported. Two multivariable logistic regression models were constructed to identify independent predictors of HPV and HPV vaccine awareness, adjusting for all covariates. Multicollinearity was evaluated using variance inflation factors (VIF < 2.5), and model fit was assessed using Hosmer-Lemeshow goodness-of-fit tests and pseudo-R² statistics. Given the hypothesis-driven nature of the study and the limited number of planned comparisons, no formal multiple-comparison correction was applied; statistical significance was set at p < 0.05.

## Results

Participant characteristics

After applying inclusion and exclusion criteria, the final analytic sample comprised 1,105 adults aged 18-44 years with self-reported depression and/or anxiety (Figure 1). Most respondents were female (64.1%), aged 18-34 years (56.4%), White (68.2%), and urban residents (86.5%). Approximately one-third (33.5%) held a college or postgraduate degree, and 34.8% reported household income ≥ $75,000. A total of 63.5% had a regular healthcare provider. Weighted prevalence estimates showed that 85.4% of participants were aware of HPV, while 75.9% were aware of the HPV vaccine.

Table 1 presents the weighted sociodemographic and clinical characteristics of the study sample and their bivariate associations with HPV and HPV vaccine awareness.

**Table 1:** Participant characteristics and bivariate associations with HPV and HPV vaccine awareness Data represented as N (%). The χ² test is used to assess the association between categorical variables and HPV/vaccine awareness. Statistical significance is defined at p < 0.05. HPV, human papillomavirus

Variable	N (%)	HPV awareness N (%)	χ², p-value	HPV vaccine awareness N (%)	χ², p-value
Sex					
Male	396 (35.9)	314 (79.2)	χ² = 6.88, p = 0.009	254 (64.1)	χ² = 25.32, p < 0.001
Female	709 (64.1)	632 (89.1)	-	602 (84.8)	-
Age					
18-34	623 (56.4)	536 (86.0)	χ² = 0.08, p = 0.776	474 (76.1)	χ² = 0.02, p = 0.900
35-44	482 (43.6)	410 (85.0)	-	382 (75.6)	-
Education					
High school or less	369 (33.4)	259 (70.2)	χ² = 46.7, p < 0.001	188 (50.8)	χ² = 76.3, p < 0.001
Some college	364 (33.1)	315 (86.5)	-	273 (75.0)	-
College graduate or higher	372 (33.5)	372 (92.6)	-	327 (87.9)	-
Race/ethnicity					
White	753 (68.2)	659 (87.5)	χ² = 9.4, p = 0.024	582 (77.3)	χ² = 17.2, p < 0.001
Black/African American	157 (14.2)	127 (81.0)	-	111 (70.7)	-
Hispanic	138 (12.5)	100 (72.5)	-	81 (58.7)	-
Other	57 (5.1)	60 (87.0)	-	46 (75.0)	-
Comorbidities					
None	522 (47.3)	439 (84.1)	χ² = 6.11, p = 0.047	395 (75.7)	χ² = 2.34, p = 0.311
1	373 (33.7)	323 (86.5)	-	285 (76.4)	-
≥2	210 (19.0)	184 (87.6)	-	176 (76.3)	-

Table 2 summarizes the multivariable logistic regression models identifying independent predictors of HPV and HPV vaccine awareness after adjusting for covariates. 

**Table 2:** Multivariable logistic regression for HPV and HPV vaccine awareness Weighted logistic regression adjusted for all covariates (age, sex, race/ethnicity, education, income, marital status, health insurance, regular provider, smoking, comorbidities, and residence). Statistical significance set at p < 0.05. Data represented as adjusted odds ratios (aOR) with 95% confidence intervals (CI). HPV, human papillomavirus

Variable	HPV awareness aOR (95% CI)	p-value	HPV vaccine awareness aOR (95% CI)	p-value
Sex (male vs. female)	0.40 (0.18-0.92)	0.003	0.23 (0.12-0.43)	<0.001
Age (35-44 vs. 18-34)	0.94 (0.55-1.61)	0.82	0.98 (0.60-1.59)	0.94
Education				
Some college vs HS or less	2.71 (1.20-6.12)	0.017	3.89 (1.66-9.08)	0.002
College+ vs. HS or less	5.35 (1.66-17.30)	0.005	5.80 (2.23-15.12)	<0.001
Race/ethnicity				
Black vs. White	0.70 (0.34-1.44)	0.335	0.82 (0.44-1.53)	0.54
Hispanic vs. White	0.41 (0.16-0.98)	0.048	0.30 (0.14-0.65)	0.003
Comorbidities (≥2 vs. 0)	3.30 (1.10-9.91)	0.033	1.05 (0.48-2.30)	0.908

## Discussion

This study provides important insights into the prevalence and correlates of HPV and HPV vaccine awareness among a nationally representative sample of U.S. adults aged 18-44 years with self-reported depression and/or anxiety [11]. While the majority of this population reported awareness of HPV (85.4%), awareness of the HPV vaccine was notably lower (75.9%). These rates are comparable to, though slightly below, those observed in the general U.S. adult population, indicating that awareness remains suboptimal in this mentally vulnerable subgroup, particularly regarding the vaccine [2].

A key finding of this study is the strong association between educational attainment and awareness of both HPV and the HPV vaccine. Participants with higher education consistently demonstrated significantly greater odds of awareness compared to those with a high school education or less. This aligns with prior research linking education to health literacy and knowledge of preventive services, including HPV vaccination [3,12,13]. These findings highlight the need for public health communication strategies that are accessible and comprehensible across all educational levels, especially when addressing complex health topics such as cancer prevention through vaccination.

Significant gender disparities were also observed, with males reporting substantially lower awareness of both HPV and the HPV vaccine compared to females. This echoes prior studies in the general population [14,15] and may be explained, in part, by the historical emphasis of HPV vaccination campaigns on females due to the initial focus on preventing cervical cancer [16]. Despite current gender-neutral recommendations and evidence of HPV-related disease burden in males, awareness remains disproportionately low. Public health efforts should therefore consider gender-specific strategies to enhance vaccine messaging among men.

Racial and ethnic disparities were another critical finding, particularly among Hispanic participants, who had significantly lower odds of awareness compared to White participants, even after adjusting for other sociodemographic factors [16,17]. These findings align with existing literature on lower HPV knowledge and vaccine uptake in Hispanic populations, which has been attributed to factors such as language barriers, cultural norms, and differential access to healthcare and health information. These findings underscore the importance of culturally tailored and linguistically appropriate interventions to address these persistent gaps.

Interestingly, participants with two or more medical comorbidities had higher odds of HPV awareness but not HPV vaccine awareness. This may suggest that individuals with greater healthcare utilization are more likely to encounter general health information, including HPV-related topics. However, these interactions may not consistently include specific discussions about vaccination, which could explain the discrepancy [18]. Further research is warranted to explore how preventive care conversations unfold among patients with multiple chronic conditions.

The study’s focus on individuals with self-reported depression and/or anxiety is particularly meaningful. Although we did not compare this group directly to non-depressed individuals, our findings indicate that within this population, subgroups such as males, those with lower education, and Hispanic individuals face compounded disadvantages in HPV-related awareness [10,11]. Mental health conditions can impair information processing and reduce engagement with preventive care [18], which suggests a valuable role for mental health professionals and primary care providers in bridging these gaps. Brief, integrated counseling on vaccine-preventable diseases during mental health visits may be an effective strategy.

Finally, the lack of significant associations for variables such as age (within the 18-44 range), marital status, rural/urban residence, insurance status (in HPV awareness), having a regular provider, and smoking status may indicate that, within this population, sociodemographic and clinical predictors such as education, gender, and ethnicity are more influential. Nonetheless, regular provider access and health insurance are generally associated with improved preventive care utilization [1], and their non-significant effects here merit deeper investigation, particularly in the context of mental health-related healthcare barriers.

Limitations

This study has several limitations that should be considered when interpreting the findings. First, the cross-sectional design limits the ability to draw causal inferences between the identified predictors and HPV or HPV vaccine awareness. Second, as all data were self-reported, recall and social desirability bias may have influenced participants’ responses, particularly concerning mental health diagnoses and HPV or vaccine awareness, which could lead to possible over- or under-reporting. Third, while HINTS provides a nationally representative dataset, our analysis focused on adults aged 18-44 years with self-reported depression and/or anxiety. Thus, the findings may not be generalizable to all individuals with these conditions, to those with undiagnosed or untreated mental-health disorders, or to older adults. The measure of depression and/or anxiety was based on whether a participant had “ever been told” by a healthcare professional, which does not capture current severity, recency, or treatment status. Fourth, although the HINTS response rates (30-36%) are consistent with other national surveys, the potential for non-response bias remains. Fifth, the study measured awareness using simple binary (“ever heard of”) items, which provide limited insight into the depth or accuracy of HPV knowledge, such as understanding of transmission, vaccine efficacy, or cancer-prevention benefits. Sixth, while a comprehensive set of covariates was included, unmeasured confounders-such as health literacy, preferred information sources, and provider-patient communication quality-may still influence awareness outcomes. Finally, data collection occurred between 2017 and 2020, prior to the COVID-19 pandemic, and may not reflect post-pandemic changes in preventive-care engagement or vaccine communication trends.

## Conclusions

This study underscores that while overall awareness of HPV is relatively high among U.S. adults aged 18-44 with self-reported depression and/or anxiety, significant gaps remain in HPV vaccine awareness, particularly among key demographic subgroups. Lower educational attainment, male sex, and Hispanic ethnicity were consistently associated with lower awareness of both HPV and the HPV vaccine. In contrast, individuals with multiple comorbidities exhibited greater awareness of HPV, though this did not extend to vaccine awareness.

These findings highlight the urgent need for targeted public health efforts to improve awareness and health literacy surrounding HPV prevention in this potentially vulnerable population. Interventions should prioritize culturally appropriate, accessible educational materials and outreach strategies tailored to populations with historically lower awareness, including males, Hispanic individuals, and those with lower levels of education. Healthcare providers may play a crucial role in disseminating accurate, evidence-based information about HPV and the benefits of vaccination. Integrating brief counseling or educational prompts into routine mental health care could be an effective strategy to bridge awareness gaps.

Closing these knowledge disparities is essential for promoting informed decision-making and ensuring equitable access to preventive services such as the HPV vaccine. Further research should explore the depth and accuracy of HPV-related knowledge in this population, identify barriers to vaccine awareness and uptake, and assess the effectiveness of targeted communication interventions designed to enhance HPV prevention among individuals with depression and anxiety. Additionally, interventional studies are warranted to empirically test the effectiveness of integrating HPV education within mental health and primary care settings, as the proposed strategies are currently theoretical.
